# Population Genetics of SARS-CoV-2: Disentangling Effects of Sampling Bias and Infection Clusters

**DOI:** 10.1016/j.gpb.2020.06.001

**Published:** 2020-07-12

**Authors:** Qi Liu, Shilei Zhao, Cheng-Min Shi, Shuhui Song, Sihui Zhu, Yankai Su, Wenming Zhao, Mingkun Li, Yiming Bao, Yongbiao Xue, Hua Chen

**Affiliations:** 1 Beijing Institute of Genomics, Chinese Academy of Sciences, Beijing 100101, China; 2 China National Center for Bioinformation, Beijing 100101, China; 3 University of Chinese Academy of Sciences, Beijing 100049, China; 4 Center for Excellence in Animal Evolution and Genetics, Chinese Academy of Sciences, Kunming 650223, China

**Keywords:** COVID-19, SARS-CoV-2, Phylogenetic divergence, Infection cluster, Sampling bias

## Abstract

A novel RNA virus, the severe acute respiratory syndrome coronavirus 2 (**SARS-CoV-2**), is responsible for the ongoing outbreak of coronavirus disease 2019 (**COVID-19**). Population genetic analysis could be useful for investigating the origin and evolutionary dynamics of COVID-19. However, due to extensive **sampling bias** and existence of **infection clusters** during the epidemic spread, direct applications of existing approaches can lead to biased parameter estimations and data misinterpretation. In this study, we first present robust estimator for the time to the most recent common ancestor (TMRCA) and the mutation rate, and then apply the approach to analyze 12,909 genomic sequences of SARS-CoV-2. The mutation rate is inferred to be 8.69 × 10^−4^ per site per year with a 95% confidence interval (CI) of [8.61 × 10^−4^, 8.77 × 10^−4^], and the TMRCA of the samples inferred to be Nov 28, 2019 with a 95% CI of [Oct 20, 2019, Dec 9, 2019]. The results indicate that COVID-19 might originate earlier than and outside of Wuhan Seafood Market. We further demonstrate that genetic polymorphism patterns, including the enrichment of specific haplotypes and the temporal allele frequency trajectories generated from infection clusters, are similar to those caused by evolutionary forces such as natural selection. Our results show that population genetic methods need to be developed to efficiently detangle the effects of sampling bias and infection clusters to gain insights into the evolutionary mechanism of SARS-CoV-2. Software for implementing VirusMuT can be downloaded at https://bigd.big.ac.cn/biocode/tools/BT007081.

## Introduction

The severe acute respiratory syndrome coronavirus 2 (SARS-CoV-2), a novel RNA virus of the Coronaviridae family, caused an outbreak of coronavirus disease 2019 (COVID-19) in China in late December 2019, and has been rapidly spreading to more than 214 countries and areas since then [1], [2]. COVID-19 is the third pandemic caused by coronavirus in the last 20 years; and it has resulted in more than 4,993,470 infections and claimed nearly 327,738 lives as of May 22, 2020, exceeding any other epidemic caused by betacoronaviruses in the human history, for example, SARS in 2002–2003 and the Middle East respiratory syndrome (MERS) in 2012 (https://www.ecdc.europa.eu/en/2019-ncov-background-disease). Among the extensive studies conducted on COVID-19, one essential question is to trace the origin and transmission between humans, shedding light on the molecular mechanism underlying epidemiological and pathological characteristics of the virus.

Population genetic methods are often used to reconstruct evolutionary history of viral infectious diseases, which supplements our knowledge of epidemic or pandemic dynamics [3], [4], [5], [6]. High evolutionary rates, which are typical of RNA viruses (10^−4^–10^−3^ nucleotide substitutions per year) [7], and large genome size of betacoronaviruses (~ 30 kb) leave sufficient amount of genomic polymorphisms within the time frame of epidemic outbreaks [8]. By interrogating the genomic data sampled at different time points of outbreaks, it is possible to estimate fundamental parameters of the evolutionary process, including evolutionary rate, population expansion rate, and the time when all sampled virus strains shared the most recent common ancestor (MRCA), and to test the different hypotheses of evolutionary mechanism. There are limitations on directly applying existing population genetic approaches to estimate the viral evolutionary history. First, virus samples are often collected by different agencies during the process of infectious outbreak, which may incur spatial and temporal sampling biases. Second, transmission of infectious diseases is commonly seen to happen in infection clusters or outbreak clusters, *i.e.*, a sudden burst of infected cases in the same place around the same time. One example of an infection cluster is the COVID-19 outbreak in the Diamond Princess cruise [9]. Both sampling bias and presence of infection clusters cause genomic polymorphism patterns similar to those generated by evolutionary effects, such as natural selection [10], [11]. Most population genetic methods are under the assumption that samples are collected uniformly and randomly from one or multiple populations. A direct application of existing population genetic approaches without taking into account of sampling bias and presence of infection clusters could lead to biased parameter estimations and data misinterpretations.

In this study, we illustrate extensive sampling biases in the SARS-CoV-2 genomic sequence data. We further investigate two other prominent polymorphism patterns found in the genomic data: a highly homozygous haplotype group or an over-sized node in the haplotype network graph, and a substantial allele frequency difference between two spatial and temporal samples (*e.g.*, the Wuhan and the non-Wuhan samples). Such data patterns are widely considered in population genetic studies as evidence of natural selection [10], [11]. Nevertheless, we propose that such patterns in the SARS-CoV-2 genomic data should result from sampling bias and presence of infection clusters during epidemic and pandemic spreads. To reduce the estimation bias, we present a robust estimator of the mutation rate and the time to MRCA (TMRCA), which disentangles the effect of sampling bias and presence of infection clusters. The performance of our proposed estimator is compared with the results from a Bayesian evolutionary analysis package, BEAST [12], via simulation studies. We subsequently apply the method to analyze 12,909 genomic sequences collected before May 4, 2020.

## Results

### Polymorphic pattern of genomic sequences

The dataset used for this study includes 12,909 genomic sequences as of May 4, 2020, of which 487 genomes are from China (15 provinces and regions) and 12,422 genomes are from 72 other countries of the world. The sampling dates range from December 24, 2019 to May 4, 2020 with a time interval of 132 days. For illustration purpose, in most of the following analysis, we focus on the subset of 756 sequences collected before March 1, 2020. After trimming off un-sequenced regions at both ends, the final alignment contains 29,599 nucleotides. There are 919 variable sites including gaps and 424 unique haplotypes among the 756 sequences. The nucleotide diversity (π) of SARS-CoV-2 is 2.36 × 10^−4^. Both Tajima’s *D* and Fu’s *Fs* values are negative (Table S1), due to highly enriched proportion of single nucleotide polymorphisms (SNPs) in singletons and rare variants. This indicates a rapid population growth or expansion of the virus population. Although π, *D*, and *Fs* are summary statistics developed for a random sample collected from a contemporary population, they do provide an informative summary of the genetic polymorphisms of the temporally collected SARS-CoV-2 sequences.

**Figure 1:**
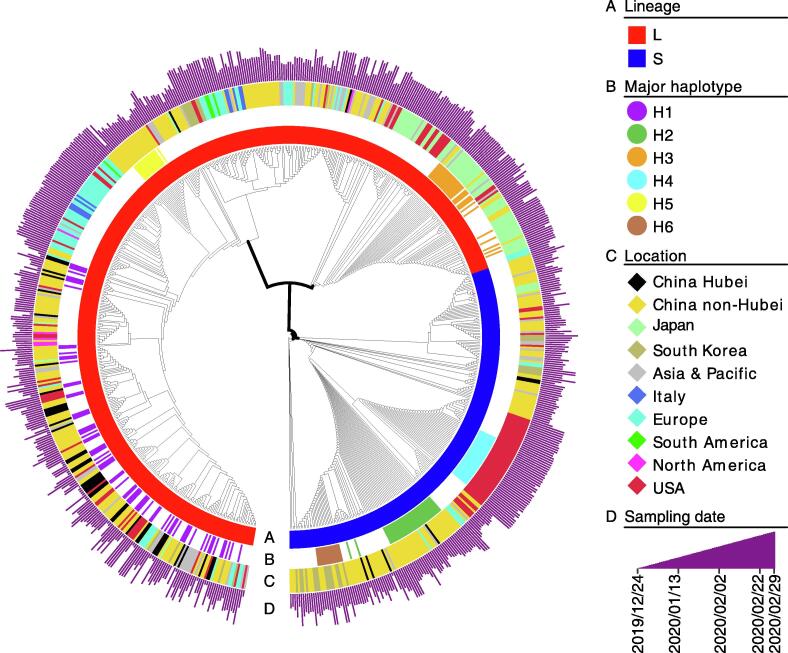
**Neighbor-joining phylogeny of 756 SARS-CoV-2 sequences collected before Mar 1, 2020 and their sampling dates and locations** The 756 SARS-CoV-2 sequences were retrieved from 2019nCoVR and GISAID. **A.** Two lineages (L and S lineages). **B.** Six major haplotypes with sequence count >10 (H1–H6). **C.** Sampling locations (China and other countries or regions). **D.** Sampling date (Dec 24, 2019 to Feb 29, 2020). Samples are color coded. 2019nCoVR, 2019 Novel Coronavirus Resource; GISAID, Global Initiative on Sharing All Influenza Data.

**Figure 2:**
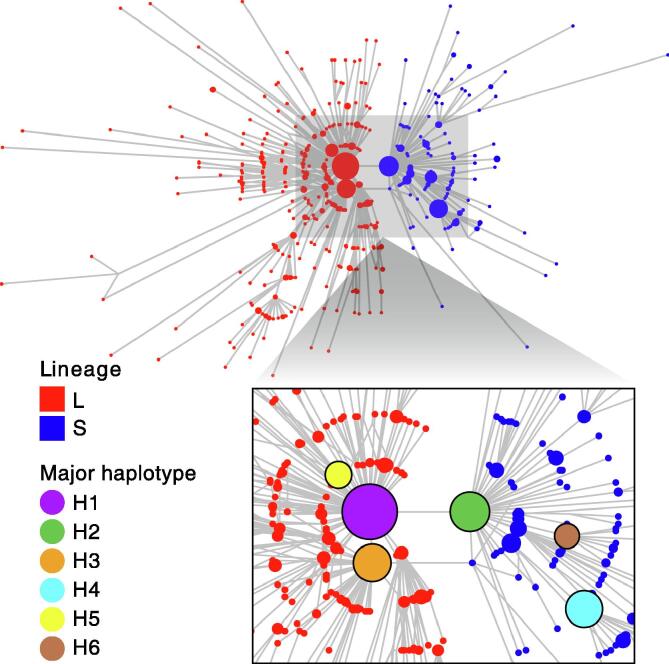
**Haplotype network of 756 SARS-CoV-2 sequences inferred with the median-joining method** The 756 SARS-CoV-2 sequences were retrieved from 2019nCoVR and GISAID. The node sizes are proportional to the counts of the sequences with the smallest node corresponding to 1 and the largest node corresponding to 68. The branch lengths are proportional to the number of mutations occurring between two haplotypes, with the shortest branch corresponding to 1 mutation and longest branch corresponding to 34 mutations. L and S lineages are denoted in red and blue, respectively. Nodes with other colors (H1–H6) represent the major haplotypes (sequence count >10). 2019nCoVR, 2019 Novel Coronavirus Resource; GISAID, Global Initiative on Sharing All Influenza Data.

A phylogeny of the 756 SARS-CoV-2 sequences is constructed using neighbor-joining approach (Figure 1). Two lineage clades, named S and L lineages, were identified in previous studies [13]. The two lineage clades are defined by one synonymous mutation occurring in open reading frame 1a (*ORF1a*) (T8782C, referring to genome position of WH-Hu-1) and one nonsynonymous mutation occurring in *ORF8* (C28144T), which leads to a replacement of serine with leucine. Among the 756 sequences, S lineage includes 110 unique haplotypes (out of 235 genomes/strains, 31.08% of total; colored in blue in Figure 1 and Figure 2), while L lineage includes 314 unique haplotypes (out of 521 genomes/strains, 68.92% of total; colored in red in Figure 1, Figure 2). Except the branch splitting the S and L lineages discussed above, many parts of the SARS-CoV-2 genealogy are highly uncertain with low support due to lack of mutations.

### Extensive spatial and temporal sampling bias

**Figure 3:**
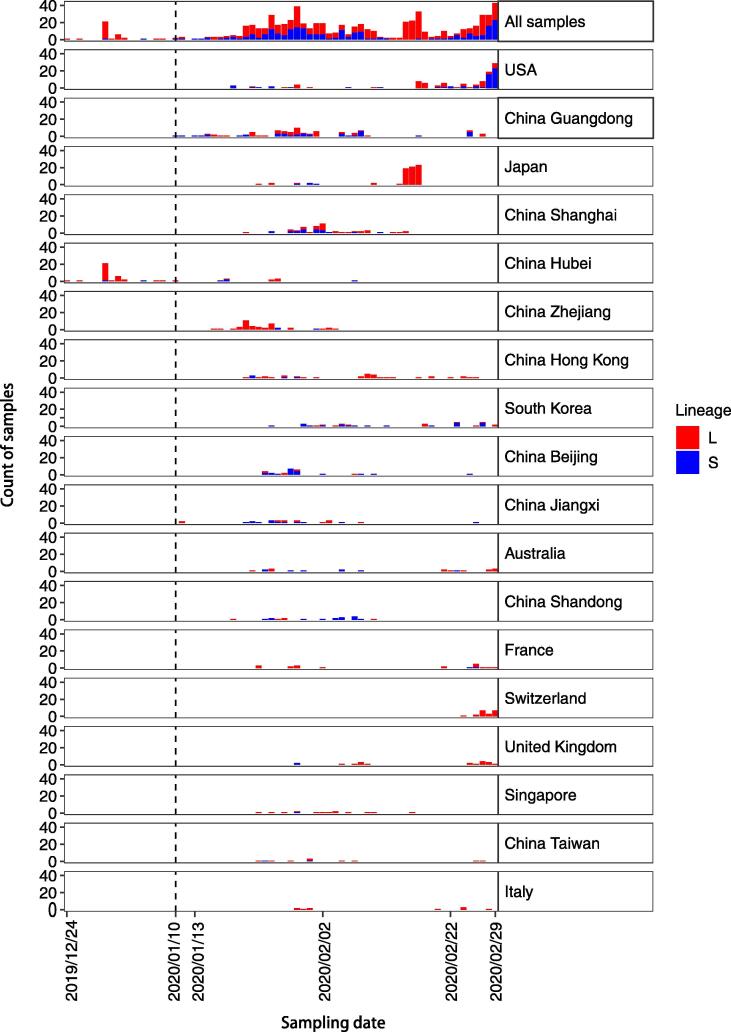
**Time-series counts of L and S lineages in samples collected from different locations before Mar 1, 2020** All sequences were retrieved from 2019nCoVR and GISAID. Only locations with >10 samples are presented. The dash line indicates Jan 10, 2020, before which almost all samples were collected from Wuhan (of Hubei Province). L and S lineages are denoted in red and blue, respectively. 2019nCoVR, 2019 Novel Coronavirus Resource; GISAID, Global Initiative on Sharing All Influenza Data.

The genomic sequences of SARS-CoV-2 were collected by multiple institutes or medical agencies at different time points of the pandemic (Figure 1 and Figure 3). Such sampling is not compliant with the population genetic assumptions on sequence samples simultaneously and randomly collected from the contemporary populations. There are at least three sources of non-ignorable sampling biases in the samples collected before March 1, 2020, which are detailed below.

First, from Dec 24, 2019 to Jan 10, 2020, 35 of the 38 sequences were collected from patients in Wuhan (Hubei Province, China; locations are all from China unless otherwise specified in the following text), 1 from Jingzhou (Hubei Province), and the 2 remaining sequences from Thailand. Most patients had direct contact with Wuhan Seafood Market (WSM), but virus sequences from other areas of Wuhan, if present, were absent from these samples. In addition, these samples were collected in a short period. The first sample date was Dec 24, 2019. On Dec 30, 2019, 21 samples were collected on the same day, among which 8 were from patients with severe symptom of the same clinical site (Jinyintan Hospital), and the rest with no information available on the collection site. Eight additional samples were collected between Jan 1, 2020 and Jan 2, 2020. The sample collection might be spatially biased toward WSM strains, since the early diagnosis protocol of COVID-19 required a direct contact of the patient with WSM. Virus samples of other areas of Wuhan might be absent or underrepresented.

Second, from Jan 10, 2020 to Feb 29, 2020, only 11 out of the 718 samples in the public databases, *i.e.*, 2019 Novel Coronavirus Resource (2019nCoVR) [14] and Global Initiative on Sharing All Influenza Data (GISAID), were collected from Wuhan. The rest of the samples were from various regions of China or other countries (Figure 3). The sample sizes ranged from 1 to 110, including 110 from USA, 91 from Guangdong Province, and 42 from Zhejiang Province (Figures 3 and S1).

Third, before Dec 24, 2019, no sequence was collected in the databases during this early stage. The first identified patient as far as we know was reported on Dec 01, 2019 by Huang and colleagues [15], without presenting the virus genomic sequences.

### Presence of multiple infection clusters during the spread of COVID-19

The genealogical relationship of the 756 genomic sequences is illustrated by a haplotype network (Figure 2). Notably, we observe multiple large nodes in the haplotype network. This pattern indicates a very common phenomenon in the spread of an infectious disease, known as an infection cluster or outbreak cluster. Infection clusters occur at a specific location with a sudden burst of infected cases during a short time interval. Genomic sequences of virus samples collected from the infected cases in an infection cluster are identical or highly similar, creating an over-sized node in the network graph.

Six nodes with sequence count >10 are presented in the zoomin (Figure 2). The H1 and H2 nodes are the major L and S lineages. Fifteen of the 68 (22.06%) H1 haplotypes were collected only from patients near WSM showing severe symptoms of COVID-19 in a short time interval (Dec 30, 2019–Jan 7, 2020), which was the initial period of COVID-19 outbreak. Samples outside of WSM were underrepresented due to the sampling bias. Similarly, another 22 of the 68 (32.35%) H1 haplotypes were also collected in a short time interval (Jan 21, 2020–Jan 25, 2020) from patients in Hangzhou (Zhejiang Province). The H3 node includes 32 samples collected during Jan 18, 2020–Feb 23, 2020, of which 26 genomic sequences were sampled during Feb 15, 2020–Feb 17, 2020 from patients on board the Diamond Princess cruise. All these sequences belong to the L lineage and are highly similar. The H4 node mostly includes samples from a nursing home and a school in the King County of Seattle (Washington, USA) during Feb 20, 2020–Feb 29, 2020. H5 consists of 16 samples with 8 collected during Jan 29, 2020–Feb 1, 2020 from the same hospital in Guangzhou (Guangdong Province). H6 includes 14 samples, among which 7 are related to a church get-together in South Korea. Apparently, the large nodes H1–H6 are nearly all related to infection clusters. Sampling bias further enhanced this pattern by over-representing them in the samples.

### Estimating the mutation rate and TMRCA

To understand how quickly SARS-CoV-2 is evolving and when the recent common ancestor of sequenced samples emerged, we propose a simple maximum likelihood method, VirusMuT, to jointly infer the mutation rate and TMRCA by constructing the likelihood function on pairwise difference between sequences. Since the virus genomes evolve and transmit among hosts during a short time interval, we make no assumptions on the virus population growth model. We assume no recombination within genomic sequences and no recurrent mutations (see “Maximum likelihood estimate of mutation rate and TMRCA” of the Materials and methods section for details).

We evaluate the performance of VirusMuT with simulated data, by checking its robustness to the sampling bias and presence of infection clusters, and compare the performance of VirusMuT with that of the commonly used method, BEAST [12]. In simulation 1 (Figure S2A and B), time-series samples composed of 300 sequences were generated from a forward simulation of virus population for 20 generations. In simulations 2 and 3, 200 additional sequences from an infection cluster were sampled. Furthermore, in simulation 3, one filtering step prior to analysis was adopted by removing identical sequences from the infection cluster. As we can see from Figure S2, both BEAST and VirusMuT show deviation from the true values in the presence of infection clusters (Figure S2C and D). However, the filtering step that down-weights sequences from the infection clusters can correct the bias in some degree (Figure S2E and F). VirusMuT overall performs better, and provides nearly unbiased inference of TMRCA and mutation rate for all three simulations, indicating its robustness to sampling bias and presence of infection clusters.

We apply VirusMuT to 12,909 genomic sequences collected before May 4, 2020 (Table 1). The mutation rate is inferred to be 8.69 × 10^−4^ per site per year with a 95% confidence interval (CI) of [8.61 × 10^−4^, 8.77 × 10^−4^]. The inferred mutation rate of SARS-CoV-2 is lower than many other RNA viruses [16], but consistent with our observation of identical genomic sequences enriched in samples collected from different places and dates. The TMRCA of the L lineage is inferred to be Dec 8, 2019 with a 95% CI of [Dec 7, 2019, Dec 9, 2019], and that of the S lineage is Dec 15, 2019 with a 95% CI of [Dec 12, 2019, Dec 18, 2019]. The TMRCA of all samples is estimated as Nov 28, 2019 with a 95% CI of [Oct 20, 2019, Dec 9, 2019].

**Table 1:** **Inferred TMRCA and mutation rate of 12,909 SARS-CoV-2 genomic sequences collected before May 4, 2020 using VirusMuT**.

**Category**	**Mutation rate (×10^−4^ site^−1^ year^−1^)**	**TMRCA**
All	8.69 [8.61, 8.77]	Nov 28, 2019 [Oct 20, 2019, Dec 9, 2019]
L lineage		Dec 8, 2019 [Dec 7, 2019, Dec 9, 2019]
S lineage		Dec 15, 2019 [Dec 12, 2019, Dec 18, 2019]

*Note*: Data are shown as mean [95% CI] TMRCA, the most recent common ancestor; CI, confidence interval.

WSM was widely considered as the source of the COVID-19 outbreak since it was first identified around late December, 2019. However, some studies claimed COVID-19 might originate at an earlier time point and outside of WSM (*e.g.*, [15]). The estimated TMRCA and the associated 95% CIs are both earlier than the outbreak time of COVID-19 in WSM. It is likely that the L and S lineages have been existing for quite a while before the outbreak.

### Pitfalls in inferring adaptive evolution of virus lineages

Another important question on virus evolutionary dynamics is to identify whether the virus genomes are under rapid adaptive evolution with the accumulation of abundant mutantations during its transmission. In population genetic analysis, a haplotype group at a high frequency and relatively low heterozygosity will exhibit an over-sized node in the network graph of a uniformly collected sample, which is often considered as a strong evidence of positive selection on the haplotype. The prominent over-sized H1 node in the haplotype network graph was identified as a signal of higher infectivity of the H1 haplotype by multiple studies [17], [18]. However, as we discussed above, over-sized nodes in the haplotype network are patterns commonly observed during virus spread due to the presence of infection clusters or severe sampling bias. Infection clusters occur mostly in places like transport hubs, nursing facilities, and schools, and usually unrelated to the infectivity or pathogenicity differences among virus lineages [19].

The other informative statistic often used to detect selection is the allele frequency difference between samples from different places or stages. We observe remarkable changes in the prevalence of L lineage during the COVID-19 pandemic (Figure S3A). For example, 35 out of 38 specimen collected before Jan 10, 2020 were from Wuhan, and 33 (94.3%) among the 35 sequences classified as L lineage; while 707 of the 718 sequences collected after Jan 10, 2020 were sampled outside of Wuhan, among which 478 (67.6%) fall into L lineage. Tang et al. [13] noticed this allele frequency difference, and hypothesized that it could be caused by different virulence of the two virus lineages and purifying selection acting on the difference. However, sampling bias in the existing data can fully explain the data pattern. The L lineage is prominently dominant in the samples collected from WSM. Since no samples from other regions in Wuhan were collected at that time, the H1 haplotype was over represented, and therefore its sample frequency did not reflect the “population” frequency in Wuhan at the early stage of the pandemic. A recent clinical study has suggested that the two lineages exhibit similar virulence and clinical outcomes [18].

Although it is a widely adopted approach in population genetics to compare the proportions of a specific allele or haplotype in different samples or to trace the allele frequency trajectories through time to evaluate the effect of natural selection, we need to be cautious about employing this approach in virus genomic analysis, because of the widespread presence of infection clusters throughout the pandemic. Indeed, most genetic polymorphism patterns, including the allele frequency spectrum and haplotype structure [20], were largely shaped by infection clusters. A direct application of existing methods in virus evolutionary study without taking into account the virus epidemiological dynamics can lead to misinterpretation.

## Discussion

Population genetic analysis of virus genomic sequences has been demonstrated to be useful to investigate the evolutionary dynamics of viruses. Tens of thousands of SARS-CoV-2 genomic sequences are publicly available for study since the outbreak of COVID-19, attracting extensive investigation. However, as we demonstrate in this paper, virus samples are different from common population genetic samples in several aspects: first, the data may be sampled massively at multiple time points; second, presence of outbreak clusters and sampling bias is common. The enrichment pattern of some subsets of haplotypes or the trend of allele frequency trajectories caused by the sampling bias are similar to that caused by evolutionary forces such as natural selection. Direct applications of existing population genetic methods may lead to biased parameter estimation or misinterpretation of evolutionary effects. Robust methods are expected to be developed by fully considering the aforementioned virus epidemiological dynamics, and improve our understanding of the evolutionary dynamics of viruses and the underlying driving forces.

## Materials and methods

### Sequence alignment, quality control, and population genetics summary

A total of 12,909 SARS-CoV-2 genomes available on May 4, 2020 were downloaded from GISAID (http://www.gisaid.org) and National Genomics Data Center (https://bigd.big.ac.cn/ncov/). The genomes were aligned using MUSCLE [21]. For a better illustration, only samples collected before March 1, 2020 were used for phylogenetic analysis. Here, a 372-bp block at the 5′-end including gaps and a 2133-bp block at the 3′-end including gaps and the poly-A tails in the alignment were trimmed out. After a pilot examination of the alignments, 141 genomes were excluded from downstream analyses due to the following sequencing quality issues: (1) presence of unusual mutations that led to outlier branches (26 genomes), (2) failure to be typed correctly to the L or S lineage (14 genomes), and (3) failure to determine the detailed sampling time (101 genomes). The final alignment includes 756 genomes composed of 29,599 sites. Population genetic summary statistics, including the number of haplotypes, gene diversity, nucleotide diversity, Tajima’s *D* [22], and Fu’s *Fs* [23], were calculated using Arlequin v3.5 [24].

### Phylogenetic and network analyses

Neighbor-joining phylogenetic tree of the 756 genome sequences was constructed using MEGA 10.1.8 with default arguments [25]. Phylogenetic relationships and mutations occurring among unique genomes were further inspected from 756 genomes through median-joining networks [26] using the Network 10 (http://www.fluxus-engineering.com/).

### Maximum likelihood estimate of mutation rate and TMRCA

The number of mutations in comparison with MRCA is assumed to follow a Poisson distribution, with the mean equal to the product of time duration (from MRCA to sampling time point) and the mutation rate. Since the sequences are sampled at different time points, TMRCA and mutation rate are identifiable. The maximum likelihood function is thus constructed below with TMRCA and mutation rate as two parameters.

Let the time duration from MRCA to the latest sampling date May 4, 2020 be T (in units of days), then the time duration from TMRCA to May 3, 2020 is T − 1 days, and so on. By assuming independent evolution among the sequences, the log likelihood function is written as

(1) $$LL\left(T,\mu \right)=\sum_{i=1}^{m}\log\left[\mathrm{poisspdf}(n_{i},\mu L\left(T-t_{i}\right))\right]$$

where *n_i_* is the Hamming distance between the i-th SARS-CoV-2 sequence and the MRCA sequence, *μ* is mutation rate (unit: /day/locus), L is the length of SARS-CoV-2 genome, *t_i_* is the time duration from sampling date to May 4, 2020 (unit: day), poisspdf is the Poisson probability mass distribution in the form $\mathrm{poisspdf}\left(k,\lambda \right)=\frac{\lambda^{k}}{k!}e^{-\lambda }$. The two parameters are then inferred by fitting the likelihood function to the data.

### Simulation of virus genomic sequence data

We used forward simulations to test the performance of BEAST v. 1.10.4 [12] and VirusMuT on estimating TMRCA and mutation rate. The genomic length was chosen to be 30,000 bp. The generation time is five days. The mutation rate is 0.001 per year per nucleotide, that is, a mean number of 0.4110 mutations occur on the genome per generation. The reproductive number (R) is 1.7. We used the Wight–Fisher model to simulate forward in time and assumed no recombination among virus strains. In each generation, the number of decedents of every virus strain was generated from a Poisson distribution with the mean R value of 1.7, and the number of mutation was also generated from a Poisson distribution with the mean value of 0.4110.

Three simulation datasets were generated for testing the methods. In simulation 1, we simulated the transmissions of the virus strains for 20 generations, and randomly collected time-series samples with the total size of 300 from generations 13 to 20. The sub-sample sizes of generations 13–20 were set to be 10, 10, 20, 20, 40, 40, 80, and 80, respectively. The sub-sample sizes increasing with generations in the simulations is to mimic the real datasets, of which more sequences were collected over time. In simulation 2, in addition to the same procedures performed in simulation 1, we randomly chose one strain during generation 10 as the founder genome, and simulated an additional “infection cluster” population from generations 10 to 20 using the same parameter settings. We then collected additional 200 sequences from the cluster (50 samples from generations 18 and 19, and 100 samples from generation 20). The final dataset included 300 sequences from simulation 1 and 200 sequences from the “infection cluster” population. The procedures of simulation 3 is identical to simulation 2, except that we included an additional filtering step to remove multiple sequences of the same sampling date in the samples from “infection cluster” population.

All the three simulations were repeated for 100 times. BEAST [12] and VirusMuT were applied to the simulated sequences. The inferred TMRCA and mutation rates were presented as boxplots in Figure S2.

## Code availability

The source code of VirusMuT can be downloaded at https://bigd.big.ac.cn/biocode/tools/BT007081.

## CRediT author statement

**Qi Liu:** Methodology, Formal analysis, Visualization. **Shilei Zhao:** Methodology, Formal analysis, Visualization, Software. **Cheng-Min Shi:** Formal analysis. **Shuhui Song:** Formal analysis. **Sihui Zhu:** Formal analysis. **Yankai Su:** Formal analysis. **Wenming Zhao:** Resources. **Mingkun Li:** Resources. **Yiming Bao:** Resources. **Yongbiao Xue:** Conceptualization, Writing - original draft, Supervision. **Hua Chen:** Conceptualization, Writing - original draft, Writing - review & editing, Supervision. All authors read and approved the final manuscript.

## Competing interests

The authors have declared no competing interests.

## Supplementary material

Supplementary Figure S1 **Counts of sequences belonging to L and S lineages collected in different countries and regions before Mar 1, 2020** All sequences were retrieved from 2019nCoVR and GISAID. 2019nCoVR, 2019 Novel Coronavirus Resource; GISAID, Global Initiative on Sharing All Influenza Data.

Supplementary Figure S2 **Performance of BEAST and VirusMuT on estimating MRCA, TMRCA, and mutation rate with simulated data** The inferred TMRCA (**A**) and mutation rate (**B**) using the sequence data from a single virus population (simulation 1). The inferred TMRCA (**C**) and mutation rate (**D**) using sequence data with an infection cluster (simulation 2). The inferred TMRCA (**E**) and mutation rate (**F**) using sequence data with an infection cluster, and a filtering step that down-weights samples from the infection cluster (simulation 3). MRCA, most recent common ancestor; TMRCA, time to the most recent common ancestor.

Supplementary Figure S3 **Proportion of S lineage and number of samples as a function of time A.** The proportion of the S lineage. **B.** The number of sequences increases with time. The dash line indicates the sample size on Mar 1, 2020.
