# Supplementary figures and images for: Population Genetics of SARS-CoV-2: Disentangling Effects of Sampling Bias and Infection Clusters
Source: Genomics Proteomics Bioinformatics. 2020 Jul 12;18(6):640–7. doi: 10.1016/j.gpb.2020.06.001 (PMC7354277; doi:10.1016/j.gpb.2020.06.001)

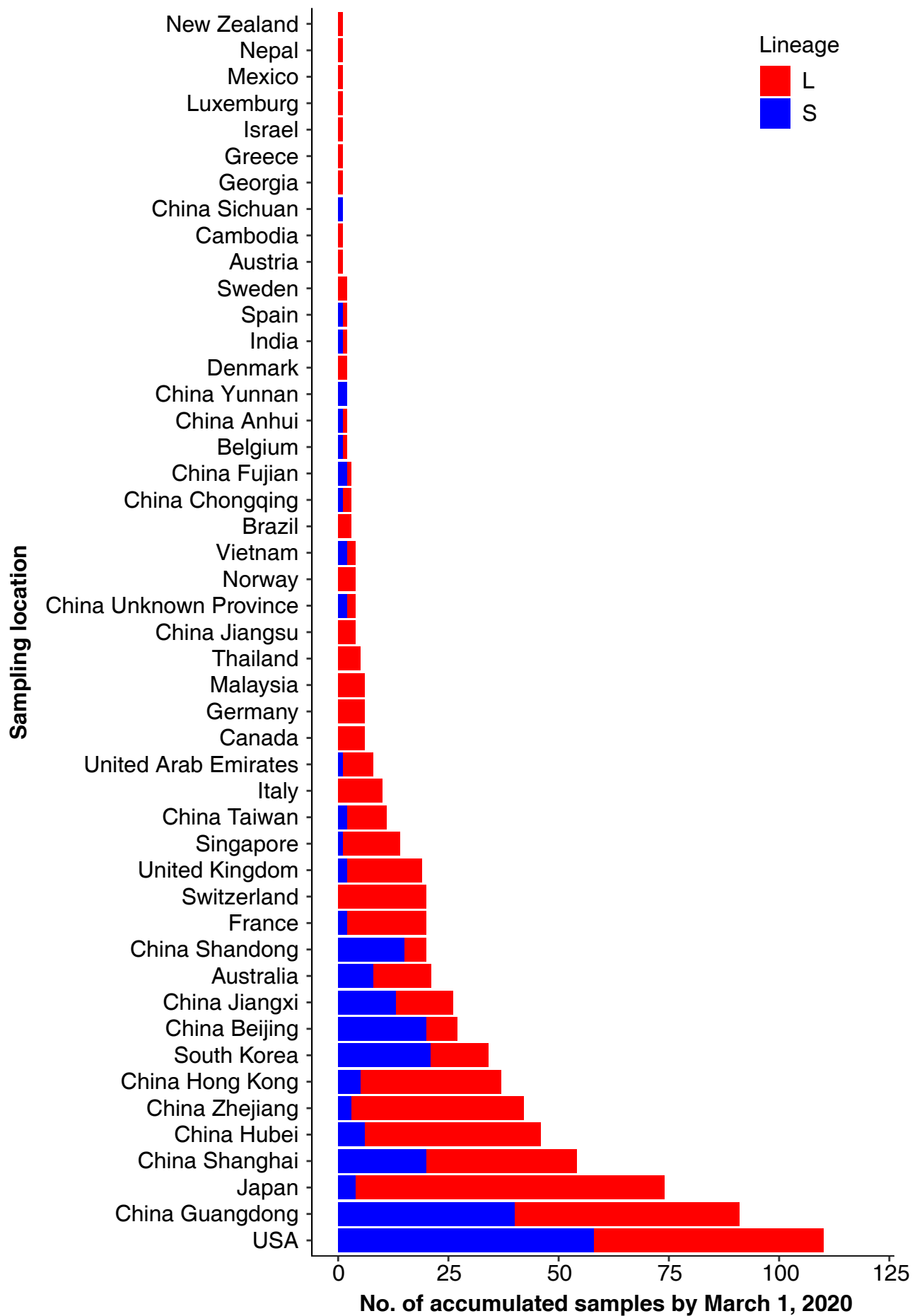

Supplement: Supplementary Figure S1 — Counts of sequences belonging to L and S lineages collected in different countries and regions before Mar 1, 2020 All sequences were retrieved from 2019nCoVR and GISAID. 2019nCoVR, 2019 Novel Coronavirus Resource; GISAID, Global Initiative on Sharing All Influenza Data. [file mmc1.pdf]

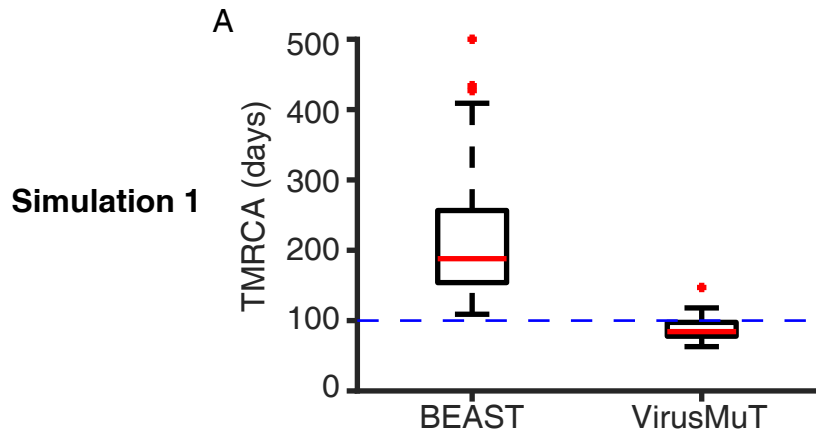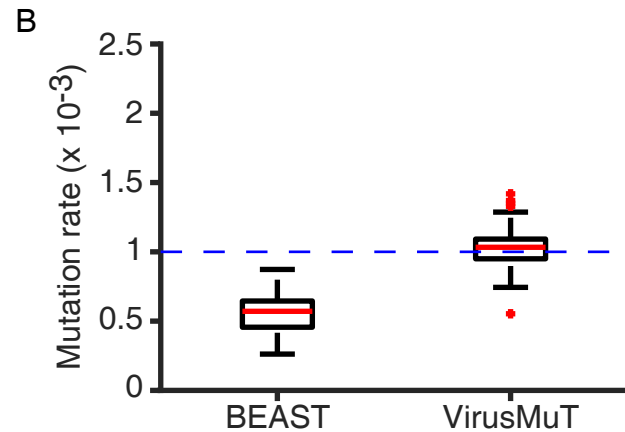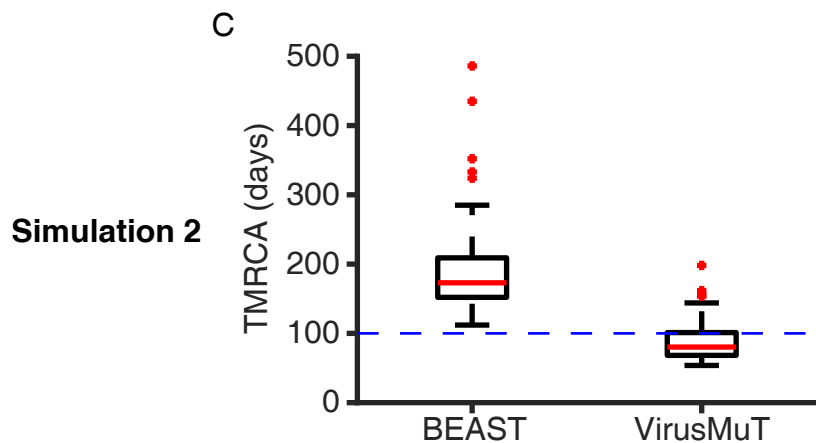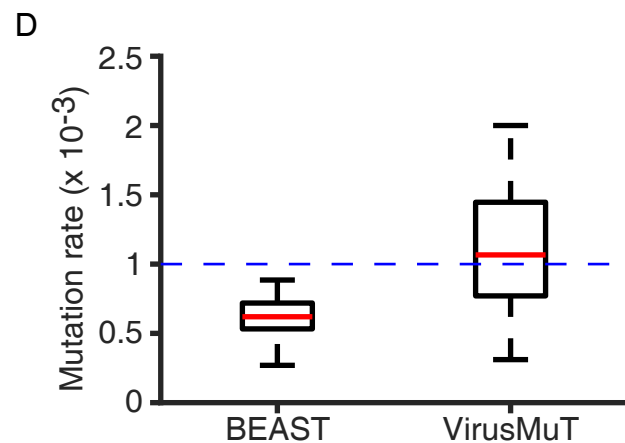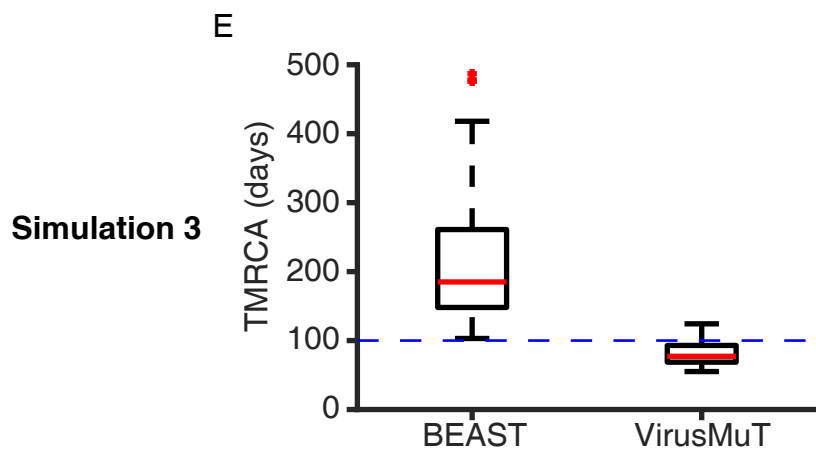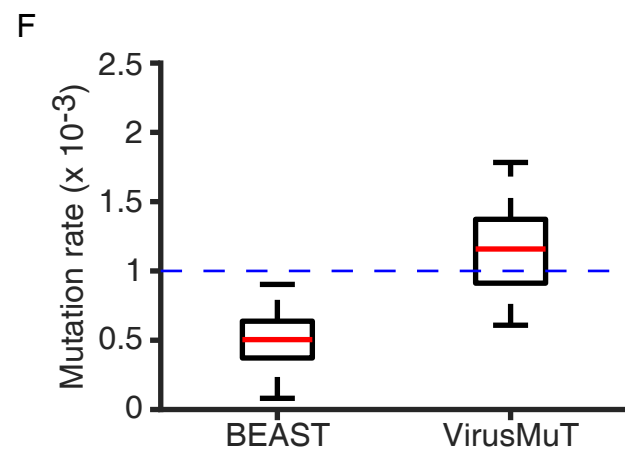

Supplement: Supplementary Figure S2 — Performance of BEAST and VirusMuT on estimating MRCA, TMRCA, and mutation rate with simulated data The inferred TMRCA (A) and mutation rate (B) using the sequence data from a single virus population (simulation 1). The inferred TMRCA (C) and mutation rate (D) using sequence data with an infection cluster (simulation 2). The inferred TMRCA (E) and mutation rate (F) using sequence data with an infection cluster, and a filtering step that down-weights samples from the infection cluster (simulation 3). MRCA, most recent common ancestor; TMRCA, time to the most recent common ancestor. [file mmc2.pdf]

**A**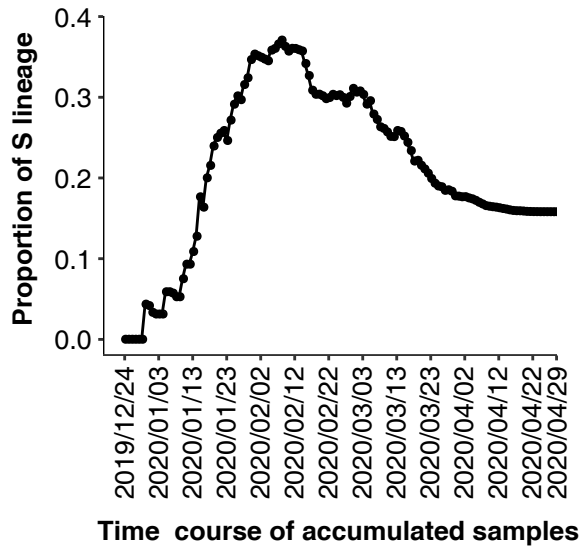**B**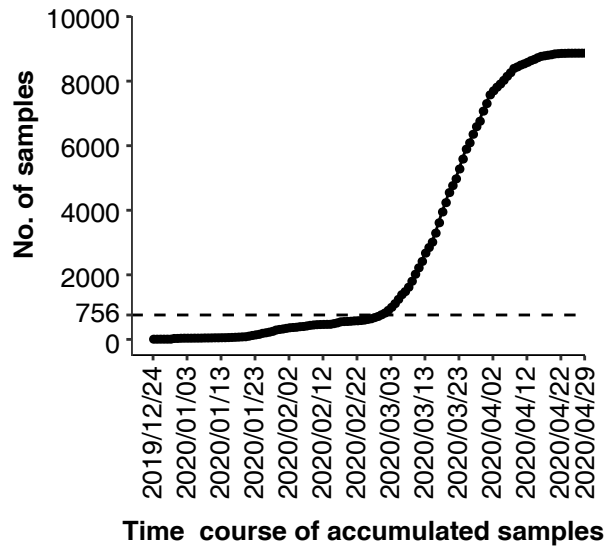

Supplement: Supplementary Figure S3 — Proportion of S lineage and number of samples as a function of time A. The proportion of the S lineage. B. The number of sequences increases with time. The dash line indicates the sample size on Mar 1, 2020. [file mmc3.pdf]
