# Supplementary material for: Population Genetics of SARS-CoV-2: Disentangling Effects of Sampling Bias and Infection Clusters
Source: Genomics Proteomics Bioinformatics. 2020 Jul 12;18(6):640–7. doi: 10.1016/j.gpb.2020.06.001 (PMC7354277; doi:10.1016/j.gpb.2020.06.001)
Supplement: Supplementary Table S1 [file mmc4.docx]

**Table S1 Summary of SARS-CoV-2 sequences collected before Mar 1, 2020**

| **Lineage** | **No. of genomes** | **No. of variable sites** | **No. of haplotypes** | **Gene diversity** | **π (× 10^-4^)** | **Tajima’s *D*** | **Fu’s *Fs*** |
| --- | --- | --- | --- | --- | --- | --- | --- |
| All | 756 | 919 | 424 | 1 | 2.36 | –2.77 (****) | –24.16 (**) |
| L lineage | 521 | 683 | 314 | 1 | 2.12 | –2.79 (****) | –24.41 (**) |
| S lineage | 235 | 261 | 110 | 1 | 1.78 | –2.66 (****) | –25.05 (****) |

*Note*: π, nucleotide diversity (average over loci). ****, *P* < 0.0001; **, 0.001 < *P* < 0.01.
